# Dataset for training neural networks in concrete crack detection: laboratory-classified beam and column images

**DOI:** 10.1016/j.dib.2025.111643

**Published:** 2025-05-21

**Authors:** Alexandre Almeida Del Savio, Ana Luna Torres, Daniel Cárdenas-Salas, Mónica Vergara Olivera, Gianella Urday Ibarra

**Affiliations:** a Carrera de Ingeniería Civil, Instituto de Investigación Científica, Universidad de Lima, Lima, Perú; b Technology Innovation Program, Carleton University, Ottawa, Canada

**Keywords:** Concrete crack detection, Neural networks, Computer vision, Structural health monitoring, Concrete beams, Concrete columns, Image classification dataset, AI in construction engineering

## Abstract

The construction industry is increasingly incorporating artificial intelligence into processes for the efficiency and accuracy of structural analysis and monitoring. However, obtaining high-quality datasets to train algorithms for detecting concrete cracks in structural components remains challenging, as such cracks normally develop over an extended period under real-world conditions. We introduce a curated dataset of 1,132 manually classified images of concrete cracks in beams and columns. These images were captured in a controlled laboratory environment using a static IP camera and annotated using the LabelImg tool. The dataset includes five object classes representing distinct cracks and failures in beams and columns and corresponding.txt files containing classification and coordinates data. This dataset is designed to facilitate developing and validating of neural network-based computer vision models for automated crack detection. It is a very useful resource for researchers in structural engineering, which enables further developments in automated structural health monitoring and contributes to the overall use of AI in the construction industry.

Specifications Table

**Table:** 

Subject	Computer Science, Engineering
Specific subject area	Artificial Intelligence, Computer Science Applications, Computer Vision and Pattern Recognition, Civil and Structural Engineering
Type of data	Raw. Images .jpg Archives .txt
Data collection	The data was acquired from one static camera, IP PTZ camera model SD10A848WA [1]. The camera was programmed using DSSExpress-Base-License software [2]. The images were manually labelled using the software tool LabelImg v.1.8.1 [3].
Data source location	All images were acquired from the Materials Laboratory of Civil Engineering Program at Universidad de Lima, Lima, Peru. Lat. -12.084307°, Long. -76.971031°
Data accessibility	The data is hosted on a public and trusted repository. Repository name: Repositorio Institucional – Universidad de Lima Data identification number: *(or DOI or persistent identifier)* Direct URL to data: https://doi.org/10.26439/ulima.datasets.19856 Instructions for accessing these data: The data is open access; anonymity is not compromised. The link to download the data set is presented in the section “Recurso(s) relacionado(s)”
Related research article	Del Savio, A. A., Luna Torres, A., Cárdenas Salas, D., Vergara Olivera, M. A., & Urday Ibarra, G. T. (2023). Detection and Evaluation of Construction Cracks through Image Analysis Using Computer Vision. Applied Sciences, 13(17), 9662. https://doi.org/10.3390/app13179662 [4]

## 1 Value of the Data

• Comprehensive Dataset: This dataset contains 1,132 high-resolution images of concrete cracks that were manually classified by five failure types. It is useful for training neural networks in structure health monitoring.
• Industry-Relevant Resource: Bridges gaps in automated crack detection by offering reproducible, laboratory-generated data, avoiding prolonged real-world collection.
• Omnidirectional Applications: This allows research in AI-based crack detection, structural maintenance, and model calibration in civil engineering.
• Open Access: It is freely available in a public repository, guaranteeing transparency and broad utility for academic and industry use.

## 2 Background

Artificial intelligence is being increasingly applied to the construction industry, enhancing efficiency and accuracy in structural analysis and monitoring. One key challenge is acquiring high-quality, diverse datasets to train AI algorithms to detect cracks in concrete since such defects usually appear after a long time under real conditions. Such an issue requires data created in controlled conditions that would allow for developing robust, reliable models.

The methodology described in [4] forms the basis for this dataset, which was developed to advance automated monitoring of construction processes by identifying cracks in structural elements. This work aligns with ongoing research efforts initiated in 2021 at the Universidad de Lima [[5], [6], [7]], which aim to provide open access, high-quality datasets to train AI algorithms for applications in the Architecture, Engineering, Construction, Operation, and Maintenance (AECO) industry. This data is of use for other researchers to improve structure monitoring and inspection methodologies through neural networks training. The high-resolution images, wide variety of crack types and detailed labelling makes it suitable for machine learning and computer vision applications and allows the development of automated development models for quick and precise structural damage evaluation.

The dataset utilizes object detection methodologies, including the YOLO v4 algorithm [4], to classify cracks in beams and columns. With this dataset, researchers can fine-tune machine learning models for better practical applications in construction. In addition, the dataset covers several gaps in previous research due to a lack of diversity and labeled data in most model validations in computer vision applications. As available high-quality concrete cracks data can be difficult to obtain, this contribution provides a reliable source for future studies.

Furthermore, the availability of this data promotes collaboration with other researchers and integration with other emerging technologies for a more efficient structural evaluation methodology.

## 3 Data Description

**Fig. 1:**
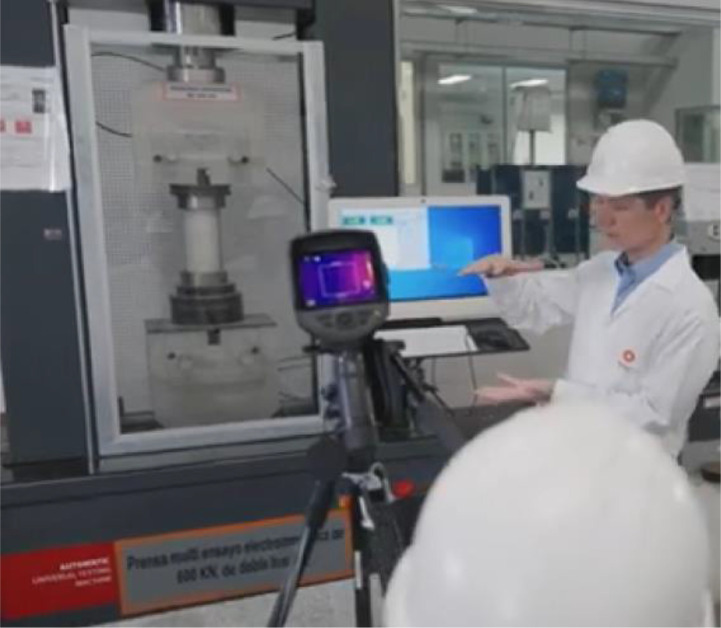
Static footage of one camera.

**Fig. 2:**
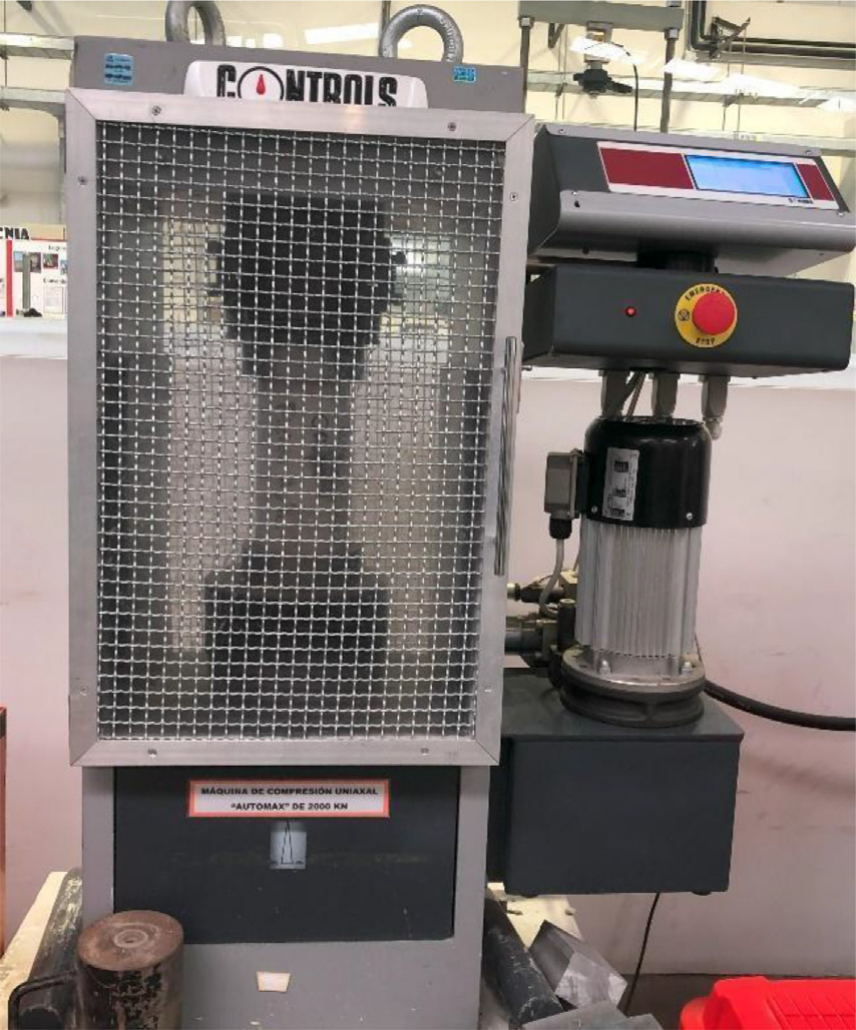
Concrete compression machines used in [1].

The dataset consists of high-resolution images of concrete columns and beams, accompanied by corresponding .txt files containing classification data and metadata. These images were captured using static footage from a single camera (Fig. 1), positioned to focus on the compression machines (Fig. 2) in a controlled environment at the Materials Laboratory of the Civil Engineering Program, Universidad de Lima. The camera was positioned between 1 m and 2 m from the object, depending on the object's size and the accessibility of the test machines. It was placed at a height of 1.30 m to 1.50 m and angled to face the object directly, ensuring the entire object was captured within the field of view. The .txt files, which document the manually classified data, were generated through meticulous annotation of the collected images [4].

Table 1 presents the classes used in the manual classification process, along with their corresponding images. The defined classes include “Beam_bending_failure,” “Column_type2_failure,” “Column_type3_failure,” “Column_type4_failure,” and “Column_type5_failure.” The classification data is provided in the accompanying classes.txt file for reference.

**Table 1:** Classes used for manual classification. Adapted from [1].

ID	Class	Object	Area of interest
0	Beam_bending_failure		
1	Column_type2_failure		
2	Column_type3_failure		
3	Column_type4_failure		
4	Column_type5_failure		

Beam_bending_failure is characterized by cracks in the center of the beam subjected to bending, generated by tensile stresses in the lower fiber, which causes a progressive crack perpendicular to the load axis, typical of bending failure in concrete beams [8].

The type of failures in concrete probes with distinct fracture patterns depends on the stress distribution. In Column_type2_failure, a well-defined cone forms at one end of the cylinder, while at the other end, the cone is incomplete, accompanied by vertical cracks at the ends, reflecting axial compression with partial cone formation. In Column_type3_failure, multiple vertical cracks appear in a columnar arrangement and poorly defined cones at the ends, suggesting uneven stress distribution. Column_type4_failure exhibits diagonal fractures that do not completely traverse the ends, indicating a combination of compressive and shear stresses. In Column_type5_failure, the fractures are located on the sides of the cylinder, usually at the top or bottom, due to uneven load distribution in specimens with unbonded heads [8].

Videos captured by the static camera were processed using a frame extraction algorithm presented in [4]. The extracted images, saved in JPG format with dimensions of 3840×2160 pixels and a resolution of 96 dpi, are displayed in the first column of Table 2. For manual classification, the LabelImg v.1.8.1 software [3] was employed to annotate and classify the relevant classes, as shown in the second column. The results, presented in the third column, include .txt files with structured data. Each file specifies: (1) the ID of the classified class (referenced in Table 1), (2) the X and Y coordinates of the annotation's starting point, and (3) the width and height of the bounding box, as detailed in Table 2. The coordinates of the bounding box are expressed in pixels, so the size and position of the object inside the image will not be affected and the measurements are absolute relative to the image resolution.

**Table 2:** Examples of selected images, IMG994.jpg and IMG543.jpg, before, during and after classification.

Original image	Image after classification	Results in .txt format
		0 0.621484 0.722222 0.066406 0.222222
		2 0.223438 0.36667 0.325000 0.538889

**Fig. 3:**
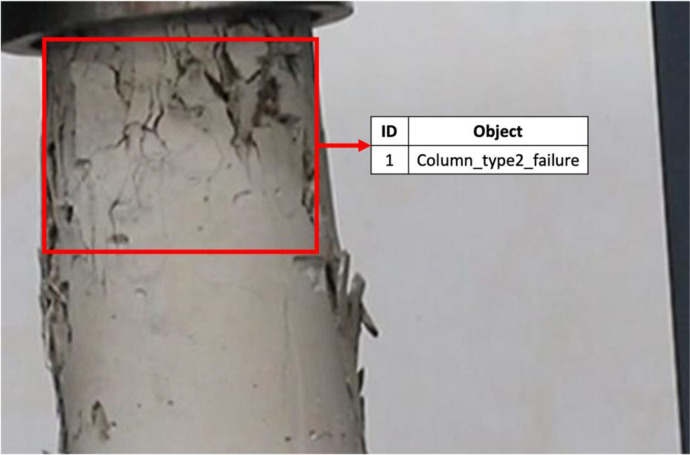
Extract of IMG994.jpg’s objects with their respective IDs.

**Fig. 4:**
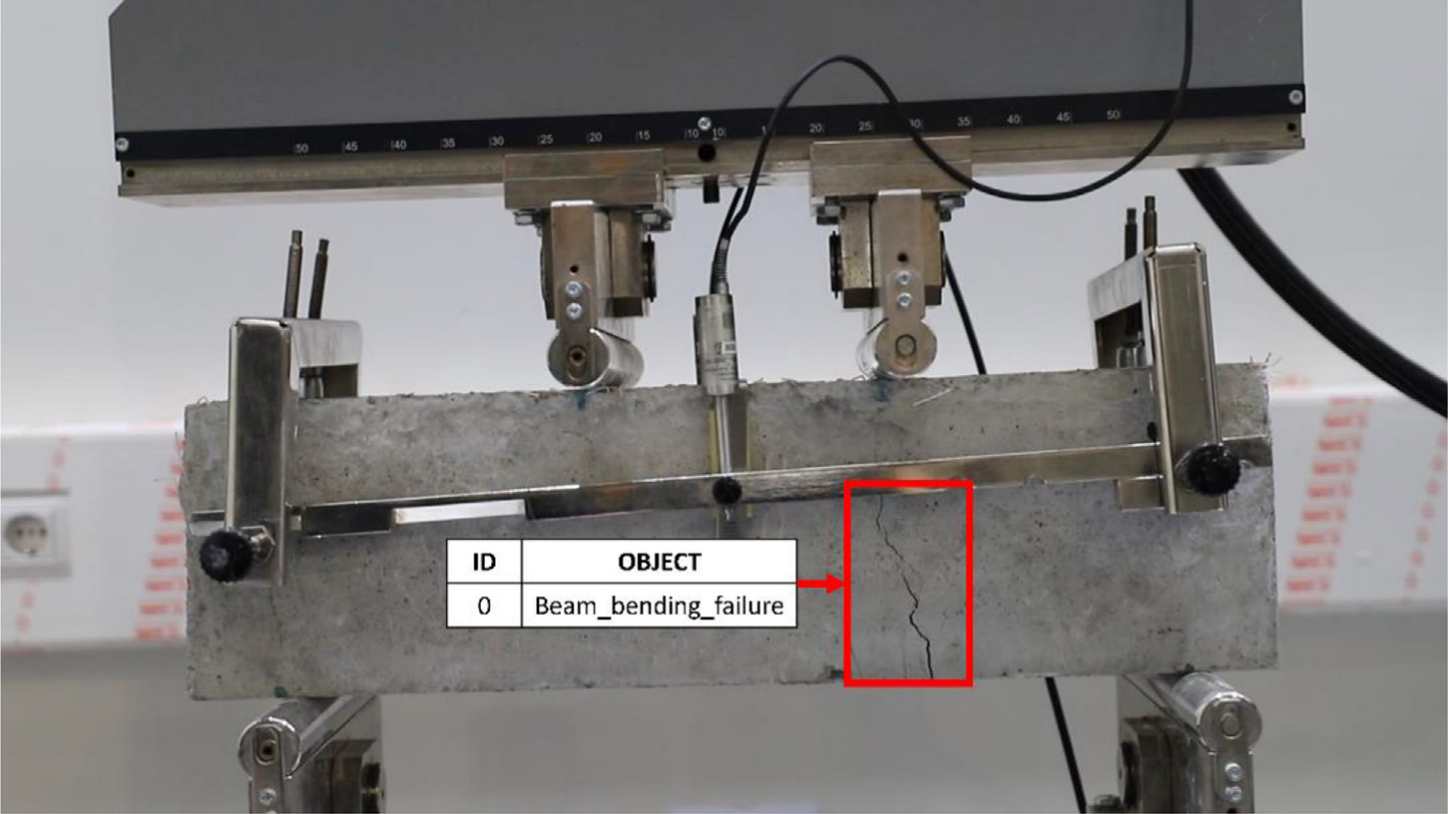
Extract of IMG543.jpg’s objects with their respective IDs.

Fig. 3, Fig. 4 show IMG994.jpg and IMG543.jpg from Table 2 where the object is linked to their respective IDs according to Table 1.

## 4 Experimental Design, Materials and Methods

### 4.1 Experimental Design

The study employs a quantitative, experimental design [9] conducted in a controlled laboratory environment. The objective was to create a high-quality dataset of concrete crack images to train and validate neural network models for automated crack detection. The experiment aimed to classify cracks on concrete beams and columns subjected to compressive forces.

The design includes the following elements:

• Purpose: To generate and classify concrete crack images for developing advanced neural network models.
• Approach: Quantitative, using image analysis for data collection and classification.
• Design: Experimental, involving controlled testing and manual classification of concrete crack images.

### 4.2 Materials

Concrete Specimens: Beams and columns fabricated with standard dimensions:

• Beams: 150 cm × 30 cm × 30 cm
• Columns: 20 cm height × 10 cm diameter
• Concrete resistance: 210 kg/cm².

Testing Equipment:

• Compression machines for applying controlled loads.
• IP PTZ camera (Model SD10A848WA) for static video recording [1].

Software Tools:

• DSS Express for video management [2].
• LabelImg v.1.8.1 for manual image annotation and classification [3].
• Frame extraction algorithm from [4].

### 4.3 Methods

Data Acquisition:

• Videos of concrete specimens under load were recorded between July 27 and August 11, 2022 (Table 3).

**Table 3:** Videos ID, duration and extracted frame quantities.

Date (YYYY/MM/DD)	Video ID	Duration (mm:ss)	Extracted frames
2022/07/27	MVI_5231.mp4	08:32	77
	MVI_5226.mp4	12:22	112
	MVI_5212.mp4	04:10	38
	IMG_0207.mp4	03:06	28
	IMG_0206.mp4	01:15	12
	IMG_0134.mp4	00:12	2
	IMG_0133.mp4	01:04	10
	IMG_0131.mp4	00:30	5
2022/08/03	IMG_0259.mp4	01:26	14
	MVI_5248.mp4	01:36	15
	MVI_5245.mp4	12:22	115
	MVI_5239.mp4	04:45	43
	MVI_5236.mp4	03:42	34
2022/08/04	MVI_5336.mp4	15:59	144
	MVI_5334.mp4	06:34	60
	MVI_5330.mp4	28:49	260
	IMG_0474.mp4	16:22	148
	IMG_0462.mp4	23:38	199
2022/08/09	MVI_5373.mp4	02:18	20
	MVI_5368.mp4	01:03	10
	MVI_5364.mp4	09:19	84
	MVI_5362.mp4	00:01	1
	MVI_5361.mp4	29:59	270
	IMG_0548.mp4	00:08	2
	IMG_0547.mp4	01:33	28
	IMG_0541.mp4	01:00	10
	IMG_0527.mp4	01:42	16
	IMG_0525.mp4	01:39	15
	IMG_0521.mp4	00:18	3
	IMG_0520.mp4	05:53	53
	IMG_0519.mp4	06:05	55
	IMG_0518.mp4	05:35	51
	IMG_0517.mp4	05:58	54
	IMG_0516.mp4	05:25	48
	IMG_0515.mp4	00:38	12
2022/08/11	MVI_5396.mp4	05:21	49
	MVI_5393.mp4	04:04	37
	MVI_5390.mp4	03:49	35
	MVI_5385.mp4	04:33	41
	MVI_5382.mp4	03:37	35
	MVI_5379.mp4	03:36	33
	MVI_5377.mp4	05:25	49
	IMG_0584.mp4	04:39	42
	IMG_0583.mp4	03:28	32
	IMG_0582.mp4	03:32	32
	IMG_0569.mp4	03:57	36
	IMG_0568.mp4	03:56	71
	IMG_0562.mp4	03:00	55
	IMG_0561.mp4	00:29	9
	IMG_0557.mp4	01:35	29
	IMG_0556.mp4	03:55	71
	IMG_0555.mp4	04:08	75
	IMG_0554.mp4	01:30	28
• The camera was positioned to capture detailed footage of cracks forming on the specimens during testing (Fig. 1).

Frame Extraction:

• Videos were processed using a frame extraction algorithm [4].
• High-resolution images (3840 × 2160 pixels, 96 dpi) were extracted, generating a total of 2,807 frames.

Image Selection and Annotation:

• From the extracted frames, 1,132 images were selected based on the visibility and relevance of cracks.
• Manual classification of images was conducted using LabelImg software. Five failure classes were defined (Table 1): Beam_bending_failure, Column_type2_failure, Column_type3_failure, Column_type4_failure and Column_type5_failure.

Data Structuring:

• Classified data were saved in .txt files with structured information:
• Column 1: Class ID (as per Table 1).
• Columns 2-5: Bounding box coordinates (X, Y, width, height).
• Metadata and images were compiled into the dataset repository.

Data Repository:

• The dataset is openly accessible via the Universidad de Lima Institutional Repository.
• Direct URL: https://hdl.handle.net/20.500.12724/19856

### 4.4 Validation and quality control

• Images and classifications were reviewed for accuracy and consistency.
• Testing conditions were monitored to ensure repeatability and reliability.

## Limitations

This study has certain limitations that may affect the generalizability and applicability of the dataset:

• Specimen Size and Configuration: The dimensions and shapes of the concrete beams and columns were limited by the laboratory equipment available. Beams were 150 cm × 30 cm × 30 cm, and columns were 20 cm in height and 10 cm in diameter, with a concrete resistance of 210 kg/cm². This restriction may not fully represent the diversity of structural elements encountered in real-world applications.
• Controlled Environment: Data collection was performed under highly controlled laboratory conditions. While this ensures consistency and precision, it may not fully replicate the variability and complexity of field conditions.
• Class Diversity: The dataset includes five predefined failure classes. Additional failure types or intermediate conditions may exist that were not captured or classified.
• Resource Constraints: The study relied on manual annotation and classification, which, while thorough, is time-consuming and limits the dataset size. Automation of this process could enable larger datasets and faster classification.

## Ethics Statement

This research did not involve human subjects, animal experimentation or social media platforms.

## CRediT authorship contribution statement

**Alexandre Almeida Del Savio:** Conceptualization, Funding acquisition, Project administration, Resources, Supervision, Validation, Writing – review & editing. **Ana Luna Torres:** Formal analysis, Resources, Validation, Visualization. **Daniel Cárdenas-Salas:** Formal analysis, Investigation, Methodology, Software, Validation. **Mónica Vergara Olivera:** Data curation, Investigation, Visualization, Writing – original draft. **Gianella Urday Ibarra:** Data curation, Investigation, Software, Writing – original draft.

## Data Availability

Repositorio Institucional – Universidad de LimaDataset of manually classified concrete cracks in images obtained from a controlled environment [Dataset] (Original data).
